# Distinct VIP and PACAP Functions in the Distal Nerve Stump During Peripheral Nerve Regeneration

**DOI:** 10.3389/fnins.2019.01326

**Published:** 2019-12-12

**Authors:** Patricia K. Woodley, Qing Min, Yankun Li, Nina F. Mulvey, David B. Parkinson, Xin-peng Dun

**Affiliations:** ^1^Faculty of Health: Medicine, Dentistry and Human Sciences, Plymouth, United Kingdom; ^2^School of Pharmacy, Hubei University of Science and Technology, Xianning, China; ^3^Department of Biology and Biochemistry, University of Bath, Bath, United Kingdom; ^4^Co-innovation Center of Neuroregeneration, Nantong University, Nantong, China

**Keywords:** VIP and PACAP, receptor expression, Schwann cells, macrophages, remyelination, inflammatory cytokines

## Abstract

Vasoactive Intestinal Peptide (VIP) and Pituitary Adenylyl Cyclase Activating Peptide (PACAP) are regeneration-associated neuropeptides, which are up-regulated by neurons following peripheral nerve injury. So far, they have only been studied for their roles as autocrine signals for both neuronal survival and axon outgrowth during peripheral nerve regeneration. In this report, we examined VIP and PACAP’s paracrine effects on Schwann cells and macrophages in the distal nerve stump during peripheral nerve regeneration. We show that VPAC1, VPAC2, and PAC1 are all up-regulated in the mouse distal nerve following peripheral nerve injury and are highly expressed in Schwann cells and macrophages within the distal sciatic nerve. We further investigated the effect of VIP and PACAP on cultured rat Schwann cells, and found that VIP and PACAP can not only promote myelin gene expression in Schwann cells but can also inhibit the release of pro-inflammatory cytokines by Schwann cells. Furthermore, we show that VIP and PACAP inhibit the release of pro-inflammatory cytokines and enhance anti-inflammatory cytokine expression in sciatic nerve explants. Our results provide evidence that VIP and PACAP could have important functions in the distal nerve stump following injury to promote remyelination and regulate the inflammatory response. Thus, VIP and PACAP receptors appear as important targets to promote peripheral nerve repair following injury.

## Introduction

The peripheral nervous system has a remarkable ability to regenerate following injury and the up-regulation of regeneration-associated genes in neurons contributes significantly to the success of repair following injury (Navarro et al., 2007; Ma and Willis, 2015). Neuropeptides such as galanin, neuropeptide Y, Vasoactive Intestinal Peptide (VIP), and Pituitary Adenylyl Cyclase Activating Peptide (PACAP) are important regeneration-associated neuropeptides for peripheral nerve regeneration (Navarro et al., 2007; Waschek, 2013). VIP is a 28 amino acid peptide, first isolated from porcine intestine, which has the ability to induce vasodilation in the canine femoral artery (Said and Mutt, 1970, 1972). PACAP is a 38 amino acid peptide and was discovered some years later, sharing 68% homology at the N-terminus with VIP (Miyata et al., 1989). Subsequent studies have revealed that both VIP and PACAP are widely distributed peptide hormones with a variety of biological activities by acting as neurotransmitters in many different organs and tissues (Harmar et al., 1998). Three receptors, named as VPAC1, VPAC2, and PAC1, have been identified for the VIP and PACAP ligands. Both VIP and PACAP can act through VPAC1 and VPAC2 whereas PAC1 selectively binds PACAP. These receptors are G-protein coupled receptors with seven transmembrane domains and the binding of VIP and PACAP to these receptors typically activates Gs and Gq/11 proteins and increases both intracellular cyclic AMP (cAMP) and inositol1,4,5-trisphosphate/diacylglycerol levels in target cells (Harmar et al., 1998; Castorina et al., 2014).

Many studies have reported that both VIP and PACAP are dramatically up-regulated in motor and sensory neurons after peripheral nerve injury (Zhou et al., 1999; Xiao et al., 2002; Armstrong et al., 2003). VIP mRNA induction in motor neurons is detectable 6 h after peripheral nerve injury and reaches a maximum up-regulation on day 7. Thereafter, the level decreases slightly but VIP expression remains high up to 30 days following injury (Armstrong et al., 2003). cDNA microarray analysis in DRG (dorsal root ganglion) at 2, 7, 14, and 28 days following injury also showed at least a 10 fold VIP up-regulation at all investigated timepoints (Xiao et al., 2002). The up-regulation of PACAP mRNA in motor neurons is also detectable 6 h after injury but PACAP peaks at 48 h with more than a 20-fold up-regulation. PACAP levels then decrease slightly from day 2 following injury, but remain more than 10-fold elevated for as long as 30 days during regeneration (Zhou et al., 1999). Previous studies have confirmed that VIP and PACAP are secreted by regenerating axons (Reimer et al., 1999), and upon release it can promote neuronal survival and axon outgrowth via receptors localized to the growth cone of regenerating axons (Lioudyno et al., 1998; Zhou et al., 1999; Armstrong et al., 2003, 2008). Reports also showed that both VIP and PACAP are up-regulated in sympathetic neurons after peripheral nerve injury, but the up-regulation is not as strong as seen in motor and sensory neurons (Zhou et al., 1999; Armstrong et al., 2003).

So far, all studies have focused on the autocrine effect of VIP and PACAP on neuronal survival and axon outgrowth during peripheral nerve regeneration (Armstrong et al., 2003; Navarro et al., 2007). However, the question remains unanswered as to why neurons dramatically up-regulate VIP and PACAP ligands and yet down-regulate their receptors, if the effects of VIP and PACAP are purely upon on regenerating axons (Zhou et al., 1999; Waschek et al., 2000). It is well known that large numbers of macrophages infiltrate into the distal nerve stump during peripheral nerve regeneration (Martini et al., 2008), and both VIP and PACAP have well characterized immunomodulatory functions on macrophages in other tissues to regulate pro- and anti-inflammatory factor expression (Delgado et al., 1999; Ganea and Delgado, 2002). There is also some evidence to show that VIP and PACAP have a direct effect on cultured Schwann cells and schwannoma cells to promote cell survival, induce laminin synthesis as well as regulating myelin protein expression (Zhang et al., 1996; Castorina et al., 2008, 2014; Lee et al., 2009).

Given the large amount of VIP and PACAP ligand released by the growth cone of regenerating axons and the down-regulation of VIP and PACAP receptors in neurons, we hypothesized that VIP and PACAP could have important paracrine effects upon Schwann cells and macrophages in the distal nerve stump to promote peripheral nerve regeneration. Therefore, in this work, we examined VPAC1, VPAC2, and PAC1 mRNA expression following mouse sciatic nerve transection injury and found that all three receptors are up-regulated in both Schwann cells and macrophages within the distal nerve stump. Next, we took *in vitro* approaches and investigated the effects of VIP and PACAP on cultured primary rat Schwann cells and mouse sciatic nerve explants. Our studies showed that VIP and PACAP could not only promote myelin gene expression in Schwann cells but also inhibited the release of pro-inflammatory cytokines by Schwann cells. Furthermore, we showed that VIP and PACAP inhibited the release of pro-inflammatory cytokines and promoted anti-inflammatory cytokine expression in sciatic nerve explants. Thus, our findings indicate that VIP and PACAP have important paracrine effects in the distal nerve stump to promote remyelination and resolve the peripheral nerve inflammatory response in order to restore nerve tissue homeostasis following repair.

## Materials and Methods

### Animals and Peripheral Nerve Surgery

All work involving animals was carried out according to Home Office regulation under the United Kingdom Animals (Scientific Procedures) Act 1986. Ethical approval for all experiments was granted by the University of Plymouth Animal Welfare and Ethical Review Board. Sprague Dawley rats and C57BL/6 mouse breeding pairs were purchased from Charles River United Kingdom Ltd. PLP-GFP mice were described before (Mallon et al., 2002; Dun et al., 2019). All animals were housed in a controlled laboratory environment (temperature 22 ± 2°C, humidity 50–60%, 12-h light/dark cycle). All animals were fed with standard rodent diet and water *ad libitum*. Two month old male and female mice were randomized. Total animals used for each experiment were stated in the figure legends. For sciatic nerve transection injury, mice were anesthetized with isoflurane, the right sciatic nerve was exposed and transected at approximately 0.5 cm proximal to the nerve trifurcation site and no re-anastamosis of the severed nerve was performed. Overlying muscle was sutured and the skin was closed with an Autoclip applier. All animals undergoing surgery were given appropriate post-operative analgesia, 0.05% bupivacaine solution, topically applied above the muscle suture before applying the surgical clip. Meloxicam (5 mg/kg) injection was given just before recovery from anesthetic. All animals undergoing surgery were given nesting material and enrichment in the cage to prevent autotomy. All animals under surgery were monitored daily. At the indicated time points post-surgery for each experiment described, animals were euthanased humanely by CO2 in accordance with United Kingdom Home Office regulations.

### VIP, PACAP and Receptor Selective Agonists, cAMP, PolyI:C and LPS

Vasoactive Intestinal Peptide (Catalog No.: 1911) and VPAC2 selective agonist (Catalog No.: Bay 55-9837) were purchased from TOCRIS. PACAP38 (Catalog No.: H-8430), VPAC1 selective agonist (Catalog No.: H-5802), and PAC1 selective agonist Maxadilan (Catalog No.: H-6734), VPAC2 selective antagonist (Catalog No.: H-7292), and PAC1 selective antagonist M65 (Catalog No.: H-6736), were purchased from Bachem. cAMP (Catalog No.: D0627), PolyI:C (Catalog No.:P0913), and LPS (Catalog No.: L3024) were purchased from Sigma.

### Primary and Secondary Antibodies

Primary antibodies against VIP (Abcam, ab78536), PACAP (Abcam, ab216627), VPAC1 (Santa Cruz, sc-30019; Abcam, ab123517), VPAC2 (Santa Cruz, sc-30020), PAC1 (Santa Cruz, sc-30018), myelin protein zero (Mpz) (Sigma, SAB2500665), myelin basic protein (Mbp) (Abcam, ab40390), neurofilament heavy chain (NF) (Abcam, ab4680), F4/80 (Abcam, ab6640), and GAPDH (EMD Millipore, MAB374) were used. Hoechst and species specific secondary antibodies conjugated with Alexa Fluor 488 or 568 dyes were purchased from Invitrogen. Horseradish peroxidase conjugated secondary antibodies for western blotting were purchased from Sigma.

### Schwann Cell Culture and Sciatic Nerve Explants

Schwann cells were prepared from sciatic nerve and brachial plexus of nine postnatal day 3 Sprague Dawley rats as previously described (Brockes et al., 1979; Dun et al., 2019). Schwann cells were cultured in low glucose (1 g/ml) DMEM containing 3% fetal bovine serum (FBS), 10 ng/ml NRG-1 (R&D, Cat No. 396-HB-050), and 2 μM forskolin (Sigma, Cat No. 344270). Intact sciatic nerve or distal sciatic nerve 10 day after transection injury were dissected out in L15 medium, the epineurium was removed under the dissection microscope and the nerves were slightly teased in L15 medium, subsequently, teased nerve segments were incubated with VIP, PACAP or receptor selective agonists in low glucose (1 g/ml) DMEM containing 5% FBS.

### Immunohistochemistry and Immunocytochemistry

Sciatic nerve and DRG samples were dissected out and fixed overnight in 4% paraformaldehyde (in PBS, PH7.2) at 4°C. Samples were then washed in PBS (3 × 10 min) and dehydrated in 30% sucrose (in PBS) overnight at 4°C. Subsequently, samples were embedded in OCT medium and sectioned on a cryostat at a thickness of 12 μm. Sections were permeabilized with 0.25% Triton X-100 plus 1% bovine serum albumin (BSA) in PBS for 45 min and then blocked with blocking buffer (3% BSA plus 0.05% Triton X-100 in PBS) for 1 h at room temperature. Sections were incubated with primary antibodies (1:100 diluted in blocking buffer) overnight at 4°C. The next day, sections were washed with PBS (3 × 10 min) and then incubated with species-specific secondary antibodies plus Hoechst dye (1:500 diluted in blocking buffer) for 1 h at room temperature. Finally, sections were washed with PBS (3 × 10 min) and mounted with Citifluor (Agar Scientific, R1320) for imaging with a Leica SP8 confocal microscope.

### Western Blot

Nerve samples were directly sonicated into 1 X SDS loading buffer. Cells were lysed in an appropriate volume of radio-immunoprecipitation assay (RIPA) buffer (50 mM Tris–HCl, pH 7.4, 0.1% SDS, 1% NP-40, 150 mM NaCl, 1 mM ethylenediaminetetraacetic acid (EDTA), 0.5% sodium deoxycholate) and phosphatase inhibitor cocktails used at 1:100 (Santa Cruz Biotechnology, sc-45045 and sc-45065) on ice, then spun down at 16,000 × *g* for 15 min at 4°C. Supernatant was transferred to new 1.5 ml microcentrifuge tubes and the protein concentration was determined using the Pierce^TM^ BCA Protein Assay Kit. An appropriate volume of sample containing 20 μg of protein was added to 4X sample buffer. Proteins were separated on 10% or 12% SDS polyacrylamide running gels and transferred onto a polyvinylidene fluoride (PVDF, 0.45 μm) transfer membrane using the wet transfer method. Membranes were blocked in 5% fat free milk in TBST (Tris buffered saline plus 0.1% Tween-20) for 1 h at room temperature. Primary antibodies were diluted (1:500) in 5% milk (in TBST) and the membranes was incubated in primary antibodies overnight at 4°C. Next day, membranes were washed in TBST (3 × 10 min) and then incubated with HRP conjugated secondary antibody (1:5000 in 5% milk, TBST) for 1 h at room temperature. After three TBST washes (10 min each), Pierce ECL western blotting substrate was added onto the membrane and incubated for 5 min to develop the chemiluminescent signal. Amersham Hyperfilm^TM^ ECL films were used to capture the intensity of the chemiluminescent signal. Exposed films were then developed in a Compact X4 automatic processor. The intensity of protein bands was quantified using the free ImageJ software available from https://imagej.nih.gov/ij/.

### mRNA Purification, cDNA Synthesis, RT-PCR and qRT-PCR

Total mRNA was extracted using a miRNeasy Mini Kit (Qiagen, 217004) and first stand cDNA was synthesized with M-MLV reverse transcriptase (Promega, M368) using random hexamer primers (Promega, C1181). RT-PCR was performed in the G-Storm GS4M, qRT-PCR was performed in the PCR LightCycler480 Real-Time PCR Instrument (Roche Applied Science) using SYBR Green I Master with primers showing in Table 1. Cross point (Cp) values were calculated by using the software of the LightCycler480 Real-Time PCR Instrument. Relative mRNA levels were calculated by the 2[-Delta Delta C(T)] method (Livak and Schmittgen, 2001) using GAPDH as a reference gene for normalization. All reactions were carried out in triplicate for statistical analysis.

**TABLE 1:** Primer sequences.

**Primers**	**Forward: 5′—3′**	**Reverse: 5′—3′**	**Accession numbers**	**Size (bp)**
Mouse VPAC1	GCCTCCACACAAGGCAAATG	GTGTTTCCAGGTAGGGCACA	NM_011703	160
Mouse VPAC2	ATAGGCGCGAGACTGAGGAA	CAACCAGCAGTAGCAGGTCA	NM_009511	135
Mouse PAC1	CCGGACCAAGTCTGGATGAC	AGCCATCCTCAGTGCAGTTC	NM_001025372	112
Mouse MCP-1	AGGTCCCTGTCATGCTTCTG	TCTGGACCCATTCCTTCTTG	NM_011333	249
Mouse TNFα	CGTCAGCCGATTTGCTATCT	CGGACTCCGCAAAGTCTAAG	NM_013693	206
Mouse ILα	GCAACGGGAAGATTCTGAAG	TGACAAACTTCTGCCTGACG	NM_010554	177
Mouse ILβ	GCCCATCCTCTGTGACTCAT	AGGCCACAGGTATTTTGTCG	NM_008361	230
Mouse TNFγ	ACTGGCAAAAGGATGGTGAC	TGAGCTCATTGAATGCTTGG	NM_008337	237
Mouse IL4	CCATATCCACGGATGCGACA	AAGCACCTTGGAAGCCCTAC	NM_021283	166
Mouse IL6	AGTTGCCTTCTTGGGACTGA	TCCACGATTTCCCAGAGAAC	NM_031168	159
Mouse IL10	GCTCTTGCACTACCAAAGCC	CTGCTGATCCTCATGCCAGT	NM_010548	112
Mouse IL13	GGCAGCATGGTATGGAGTGT	CTTGCGGTTACAGAGGCCAT	NM_008355	132
Mouse GAPDH	AAGGTCATCCCAGAGCTGAA	CTGCTTCACCACCTTCTTGA	XM_017321385	222
Rat VPAC1	GCTCCTTAAAACTGGCCCCT	TCAAACACCTCAGTGCCGTT	NM_012685	149
Rat VPAC2	GAAGGCAGAGAGGGCGATAG	CAACCAGCAGTAGCAGGTCA	NM_017238	152
Rat PAC1	AGCATTCACCCCCTTTCCTCA	GGAGAGAAGGCGAATACTGTGT	NM_001270579	175
Rat MCP-1	CAGGTCTCTGTCACGCTTCT	GGCATTAACTGCATCTGGCTG	NM_031530	87
Rat TNFα	CATCCGTTCTCTACCCAGCC	AATTCTGAGCCCGGAGTTGG	XM_008772775	151
Rat ILα	CCTCGTCCTAAGTCACTCGC	GGCTGGTTCCACTAGGCTTT	NM_017019	105
Rat ILβ	GACTTCACCATGGAACCCGT	GGAGACTGCCCATTCTCGAC	NM_031512	85
Rat IL6	AGCGATGATGCACTGTCAGA	GGAACTCCAGAAGACCAGAGC	NM_012589	106
Rat Krox20	AGGAGCAAATGATGACCGCC	CATGCCATCTCCAGCCACTC	NM_053633	185
Rat Mbp	TGTGGGGGTAAGAGAAACGC	AAGGTCGGTCGTTCAGTCAC	NM_001025291	126
Rat Mpz	ATGACCGAGGACCAATGACG	CTGTGCTCCAGAGTGGTCAG	NM_017027	102
Rat GAPDH	AGTGCCAGCCTCGTCTCATA	GGTAACCAGGCGTCCGATAC	XM_017593963	77

### Statistical Analysis

**FIGURE 1:**
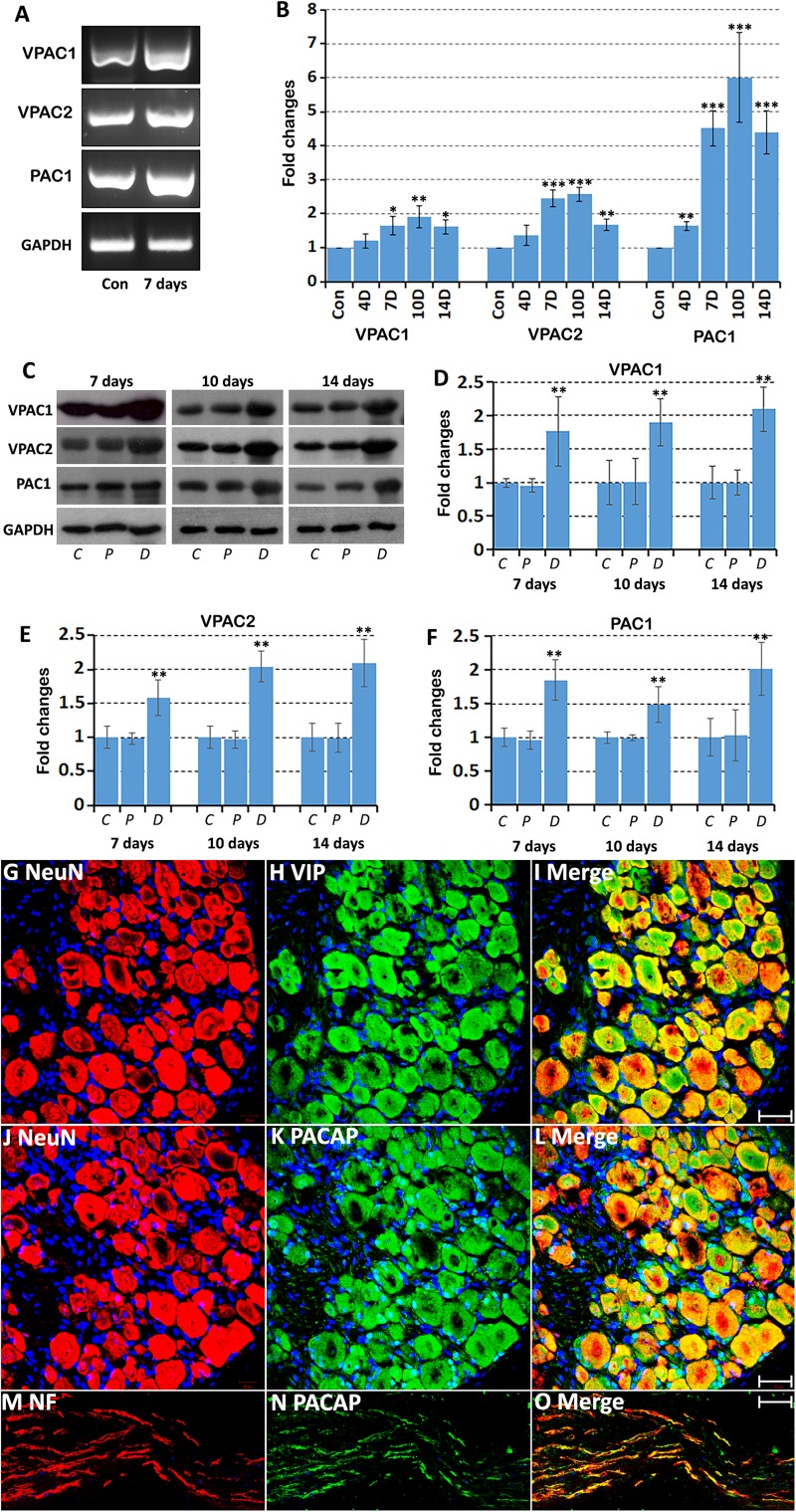
Up-regulation of VPAC1, VPAC2, and PAC1 following injury in the mouse distal sciatic nerve. **(A)** RT-PCR showing the presence of VPAC1, VPAC2, and PAC1 mRNAs in the intact (Con) mouse sciatic nerve and in the distal sciatic nerve 7 days after transection injury. **(B)** qRT-PCR showing VPAC1, VPAC2, and PAC1 mRNA up-regulation in the mouse distal sciatic nerve at 4, 7, 10 and 14 days following transection injury, *n* = 3. **(C)** Western blot showing VPAC1, VPAC2, and PAC1 protein expression in control uninjured (*C*), proximal (*P*), and distal (*D*) mouse sciatic nerve at 7, 10, and 14 days following transection injury. **(D–F)** Quantification of VPAC1 **(D)**, VPAC2 **(E)**, and PAC1 **(F)** protein levels from three independent western blot results showing VPAC1, VPAC2, and PAC1 protein up-regulation in the distal nerve stump. All samples were normalized to GAPDH and control samples were normalized to 1. ^∗^*P* < 0.05, ^∗∗^*P* < 0.01, ^∗∗∗^*P* < 0.001. **(G–I)** Double staining of Vasoactive Intestinal Peptide (VIP) with the neuronal marker NeuN showing that sensory neurons in the DRG express VIP at 7 days after sciatic nerve transection injury. **(J–L)** Double staining of Pituitary Adenylyl Cyclase Activating Peptide (PACAP) with the neuronal marker NeuN showing that sensory neurons in the DRG express PACAP 7 days after sciatic nerve transection injury. **(M–O)** Double staining of PACAP with neurofilament (NF) showing that PACAP is present in leading regenerating axons in the nerve bridge 7 days after sciatic nerve transection injury. Scale bars in I and L 20 μm. Scale bar in O 40 μm. Thirty six mice were used in **A** and **B** for RT-PCR and qPCR experiments. Twenty seven mice were used in **C-F** for western blot experiments. Three mice were used in **(G–O)** for immunostaining and three sections from each mouse were used for each staining.

Samples for Western blotting and qRT-PCR in Figures 1, 8 were prepared by grouping three nerves from three different mice together for each time point to create a pooled sample as *n* = 1, and then repeated the process using another six animals to reach *n* = 3. Therefore, we have used pooled biological replicates for the repetition of these experiments. Statistical significance was analyzed using the Student’s *t*-test and ANOVA by comparing the test groups with the control groups. All data are represented in the figures as mean value ± SEM. *P* values are indicated with single asterisk (^∗^<0.05), double asterisks (^∗∗^<0.01) and triple asterisks (^∗∗∗^<0.001) on graphs. Where graphs are not labeled with an asterisk, any differences between the test groups and the control groups were non-significant. The *n* number for each experiment has been stated in each figure legend.

## Results

### Up-Regulation of VPAC1, VPAC2 and PAC1 in the Mouse Distal Nerve Stump Following Transection Injury

We first used an RT-PCR method to detect expression of VPAC1, VPAC2, and PAC1 mRNAs in the intact mouse sciatic nerve and in the distal sciatic nerve stump 7 days following transection injury. RT-PCR result showed that VPAC1, VPAC2, and PAC1 mRNAs are all present in the intact mouse sciatic nerve and are all seemingly increased in the distal nerve stump following injury (Figure 1A). After confirming the presence of their mRNAs in the intact sciatic nerve and in the distal sciatic nerve stump, we used qRT-PCR to examine the time course of their mRNA changes at 4, 7, 10, and 14 days following sciatic nerve transection, using the contralateral intact sciatic nerve as control. VPAC1, VPAC2, and PAC1 mRNA levels are all significantly up-regulated in the distal nerve stump following injury compared to their expression in control nerves (Figure 1B). VPAC1 and VPAC2 show similar expression profiles and their expression becomes significantly elevated compared to control nerve at day 7 and peaks at day 10. PAC1 expression is significantly up-regulated at day 4 and peaks at day 10. Expression of VPAC1, VPAC2, and PAC1 begins to decrease at day 14 but remains significantly increased compared to control nerves (Figure 1B).

Next, we investigated changes in protein expression by western blot in the proximal nerve stump and in the distal nerve stump at 7, 10, and 14 days following transection injury. Consistent with their mRNA up-regulation in the distal nerve stump at day 7, 10, and 14, western blot results confirmed the up-regulation of VPAC1, VPAC2, and PAC1 proteins in the distal nerve stump at day 7, 10, and 14 post-injury (Figures 1C–F). In contrast, there were no significant changes of their protein levels in the proximal nerve stump following injury (Figures 1C–F). Thus, our RT-PCR, qRT-PCR, and western blot results not only confirmed the expression of VPAC1, VPAC2, and PAC1 in the mouse distal nerve stump but also revealed their up-regulation in the distal nerve stump following injury.

Vasoactive Intestinal Peptide and PACAP are almost undetectable in motor and sensory neurons of the adult peripheral nervous system, but both are significantly up-regulated after peripheral nerve injury (Zhou et al., 1999; Xiao et al., 2002; Armstrong et al., 2003). The up-regulation of VIP and PACAP in DRG tissue after peripheral nerve injury has been very well documented using immunostaining, *in situ* hybridization and microarray methodologies (Zhou et al., 1999; Xiao et al., 2002; Armstrong et al., 2003). To confirm VIP and PACAP up-regulation in sensory neurons of our mouse sciatic nerve transection injury model, we stained VIP and PACAP in sensory neurons of DRG tissue at 7 days after sciatic nerve transection injury. Our staining has confirmed that VIP and PACAP are expressed in sensory neurons of DRG (Figures 1G–L). We also stained for PACAP in sciatic nerve following injury and confirmed that PACAP is present in regenerating axons (Figures 1M–O). Thus, VIP and PACAP released from regenerating axons could interact with their receptors expressed in the distal nerve stump during peripheral nerve regeneration.

### Expression Pattern of VPAC1, VPAC2 and PAC1 in the Intact Mouse Sciatic Nerve

**FIGURE 2:**
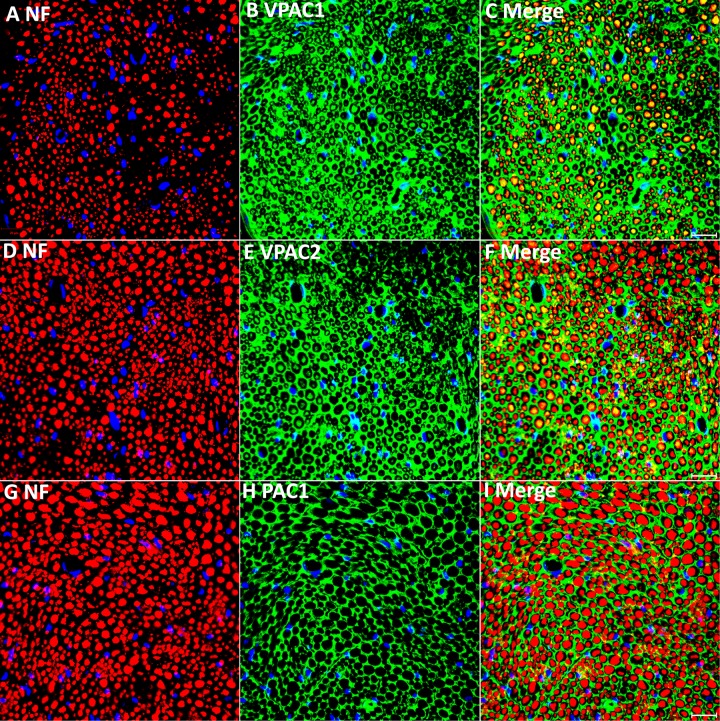
Double staining of VPAC1, VPAC2, and PAC1 with neurofilament heavy chain (NF) on transverse sections from intact mouse sciatic nerve. VPAC1 **(A–C)** and VPAC2 **(D–F)** staining both show co-localization with neurofilament (NF) but PAC1 **(G–I)** does not. Yellow color in **(C,F)** shows VPAC1 and VPAC2 co-localize with NF, respectively. VPAC1, VPAC2, and PAC1 also show positive staining in areas surrounding axons. Scale bars in **(C,F,I)** 20 μm. Three mice were used and three sections from each mouse were stained for each receptor.

**FIGURE 3:**
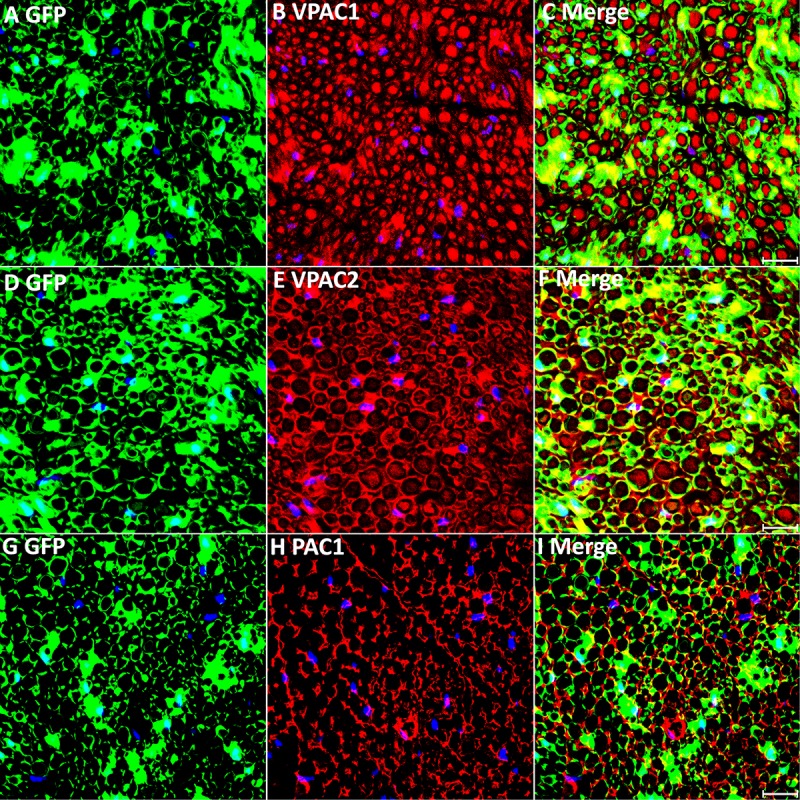
VPAC1, VPAC2, and PAC1 staining on transverse sections from uninjured sciatic nerve of PLP-GFP mice. VPAC1 **(A–C)**, VPAC2 **(D–F)**, and PAC1 **(G–I)** staining all co-localize with GFP-positive Schwann cells. VPAC1 and VPAC2 also show positive staining in axons but PAC1 **(G–I)** does not. Scale bars in **(C,F,I)** 20 μm. Three mice were used and three sections from each mouse were stained for each receptor.

Our PCR and Western blot results have revealed VPAC1, VPAC2, and PAC1 expression in intact mouse sciatic nerve. To understand more clearly the cell-specific expression of VPAC1, VPAC2, and PAC1 proteins in intact adult mouse sciatic nerve, we performed VPAC1, VPAC2, and PAC1 double staining with an axonal marker, NF, on adult sciatic nerve transverse sections. Double staining of VPAC1 with NF showed that VPAC1 strongly co-localized with NF (Figures 2A–C), indicating that VPAC1 is strongly expressed in axons of the peripheral nerve. VPAC1 also showed staining surrounding axons indicating that it is also expressed in Schwann cells (Figures 2A–C). Similarly, double staining of VPAC2 with NF showed that VPAC2 is expressed in axons and Schwann cells (Figures 2D–F). In contrast, double staining of PAC1 with NF showed that PAC1 is only expressed in Schwann cells (Figures 2G–I). To confirm the expression of VPAC1, VPAC2, and PAC1 in Schwann cells, we stained VPAC1, VPAC2, and PAC1 on sciatic nerve transverse sections from PLP-GFP mice, which label Schwann cells GFP-positive (Mallon et al., 2002; Carr et al., 2017; Dun et al., 2019). Once again, the staining showed that VPAC1 is high expressed in axons and weakly expressed in Schwann cells (Figures 3A–I). VPAC2 staining shows stronger expression in Schwann cells than in axons (Figures 3D–M). In contrast, PAC1 is only expressed in Schwann cells with clear cell membrane localization comparing to the cytoplasmic GFP signal (Figures 3G–I).

### Expression Pattern of VPAC1, VPAC2, and PAC1 in Mouse Distal Sciatic Nerve Following Injury

**FIGURE 4:**
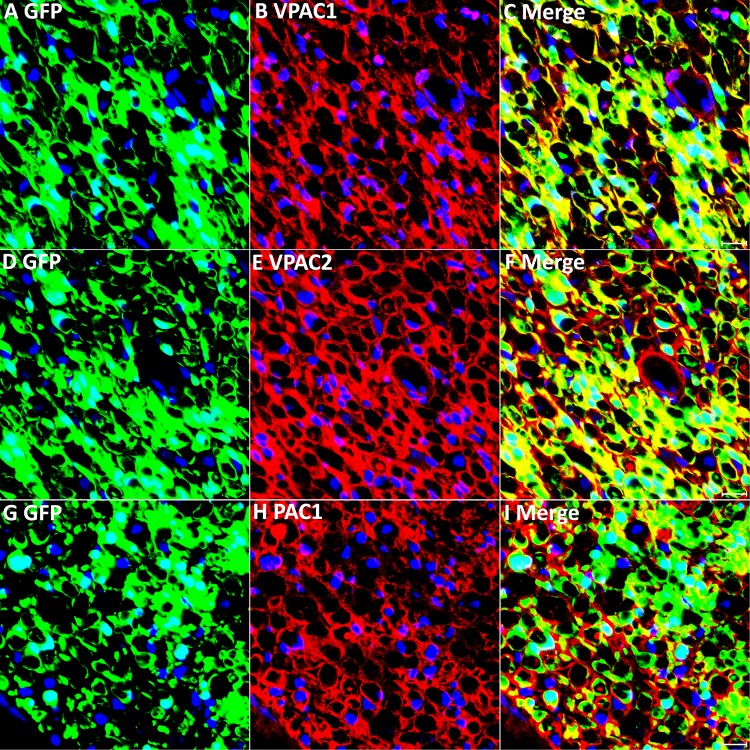
VPAC1, VPAC2, and PAC1 expression in Schwann cells of the distal nerve stump following injury. VPAC1, VPAC2, and PAC1 staining on transverse sections from distal sciatic nerve of PLP-GFP mice 7 days after transection injury. VPAC1 **(A–C)**, VPAC2 **(D–F)**, and PAC1 **(G–I)** all co-localize with GFP in Schwann cells, confirming that VPAC1, VPAC2, and PAC1 are all expressed in Schwann cells of the distal nerve stump. Scale bars in **(C,F,I)** 20 μm. Three mice were used and three sections from each mouse were stained for each receptor.

Next, we transected the sciatic nerve in PLP-GFP mice to study VPAC1, VPAC2, and PAC1 expression in Schwann cells of the distal nerve stump. Immunostaining of VPAC1, VPAC2, and PAC1 on transverse sections of distal nerve at 7 days post-injury showed that all GFP positive cells express VPAC1, VPAC2, and PAC1 receptors, confirming that VPAC1, VPAC2, and PAC1 are all still expressed in Schwann cells of the distal sciatic nerve after transection injury (Figures 4A–I). Thus, VIP and PACAP released from regenerating axons could interact with their receptors that are expressed in Schwann cells of the distal nerve stump to potentially regulate Schwann cell function during peripheral nerve regeneration.

**FIGURE 5:**
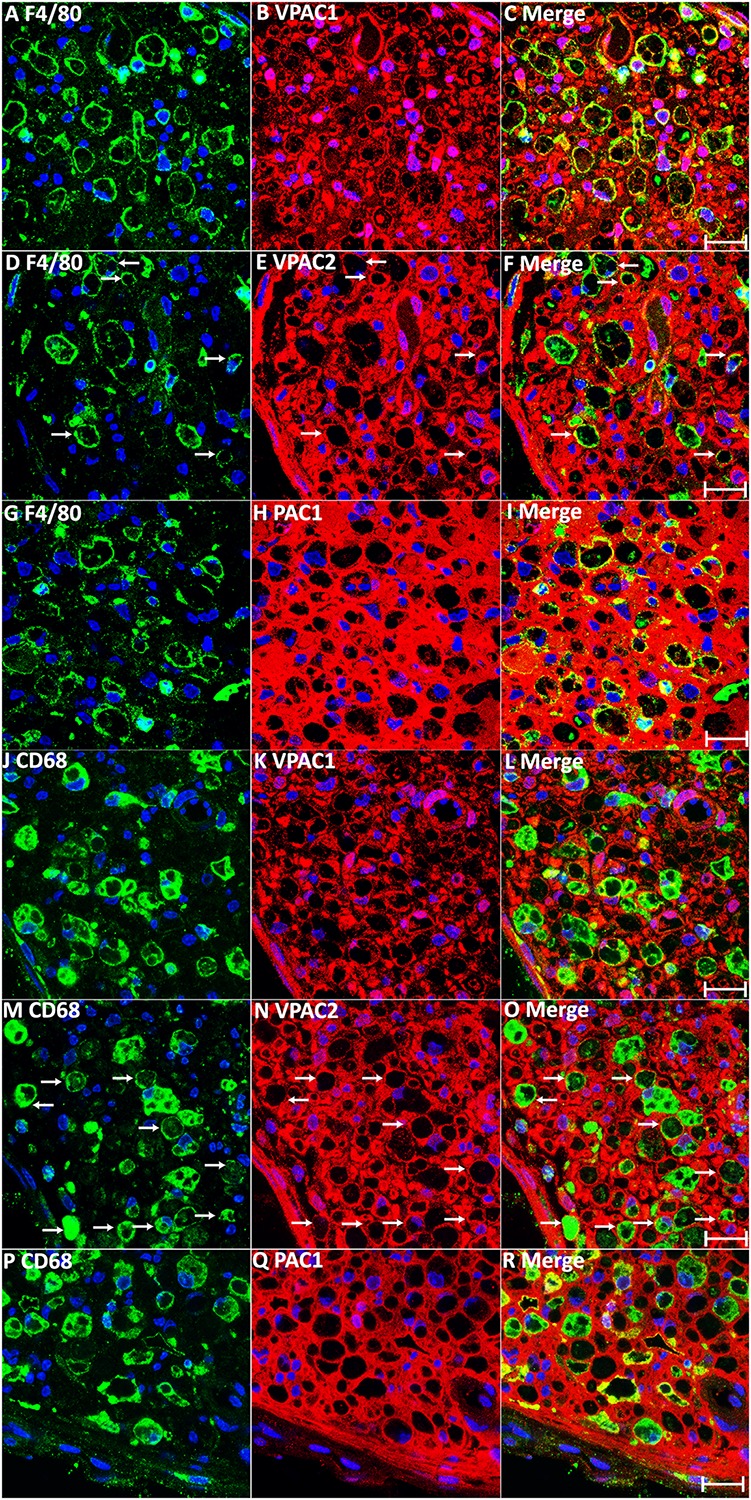
Expression of VPAC1, VPAC2, and PAC1 in macrophages of the mouse distal sciatic nerve at 7 days post-injury. **(A–I)** Double staining of VPAC1, VPAC2, and PAC1 with macrophage marker F4/80 on transverse sections from mouse distal sciatic nerve at 7 days post-transection injury. **(J–R)** Double staining of VPAC1, VPAC2, and PAC1 with macrophage marker CD68 on transverse sections from mouse distal sciatic nerve 7 days post-transection injury. VPAC1 **(A–C,J–L)** and PAC1 **(G–I,P–R)** are expressed in most macrophages of the distal nerve stump. Macrophages expressing VPAC2 are indicated by arrows in **(D–F,M–O)**. Scale bars 20 μm. Three mice were used and three sections from each mouse were stained for each receptor.

In addition to Schwann cells, macrophages are another major cell type in the distal nerve stump following injury, acting to promote peripheral nerve regeneration (Martini et al., 2008; Stierli et al., 2018). VIP and PACAP have well characterized functions in macrophages to promote anti-inflammatory cytokine expression (Ganea and Delgado, 2002), and studies in the immune system showed that macrophages express both VIP and PACAP receptors (Delgado et al., 2004; Vaudry et al., 2009). Therefore, we studied the expression of VPAC1, VPAC2, and PAC1 in macrophages of the distal nerve stump by immunohistochemistry on day 7 following mouse sciatic nerve transection injury. Double staining of VPAC1, VPAC2, or PAC1 with two well characterized macrophage markers, F4/80 and CD68, on transverse sections revealed that VPAC1 (Figures 5A–C,J–L) and PAC1 (Figures 5G–I,P–R) are expressed by most macrophages of the distal nerve stump and that some macrophages express also VPAC2 (Figures 5D–F,M–O, indicated by arrows). Thus, VIP and PACAP released from regenerating axons could also interact with receptors that are expressed in macrophages in the distal nerve stump to regulate the immune response within the nerve during regeneration.

### VIP and PACAP Increase Myelin Protein Expression in Cultured Schwann Cells

**FIGURE 6:**
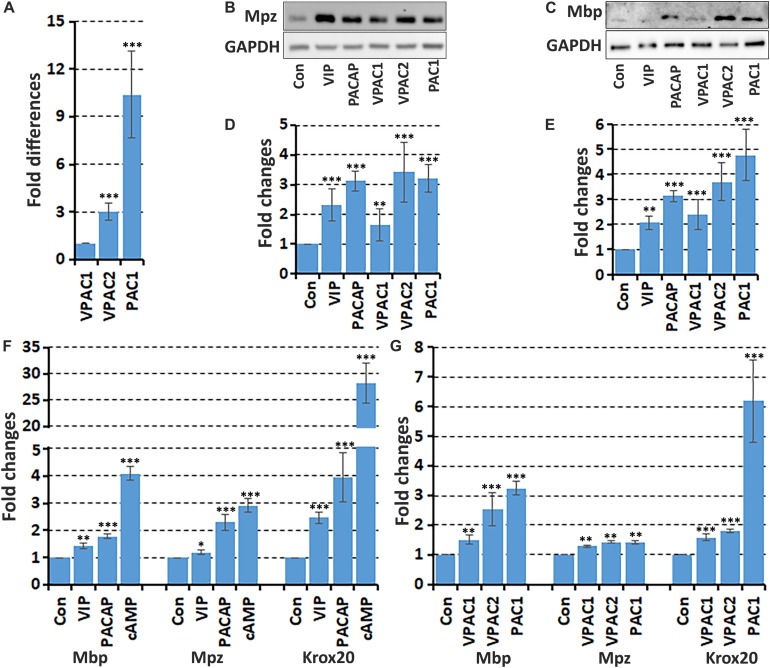
VIP and PACAP increase myelin gene expression in cultured Schwann cells. **(A)** Relative expression level of VPAC1, VPAC2, and PAC1 in cultured primary rat Schwann cells, *n* = 3. **(B,C)** Western blot results show VIP, PACAP and receptor specific agonist up-regulation of myelin protein zero (Mpz) and myelin basic protein (Mbp) protein expression in Schwann cells. **(D,E)** Quantification of Mpz and Mbp levels from three independent western blot results. **(F)** qRT-PCR data showing VIP and PACAP increase the mRNA expression of myelinating Schwann cell markers Mbp, Mpz, and Krox20 after 24 h treatment, *n* = 3. **(G)** VPAC1, VPAC2, and PAC1 receptor specific agonist treatment increases Mbp, Mpz, and Krox20 mRNA expression in Schwann cells. ^∗^*P* < 0.05, ^∗∗^*P* < 0.01, ^∗∗∗^*P* < 0.001.

It has been previously been described that VIP and PACAP treatment increases myelin protein expression in the rat RT4 schwannoma cell line (Castorina et al., 2014), but this has not been tested in primary Schwann cells. Therefore, we studied the effect of VIP and PACAP on myelin gene expression in primary rat Schwann cells. First, the relative expression levels *in vitro* of the three receptors was compared in primary rat Schwann cells by qRT-PCR. This showed that VPAC1 has the lowest expression, with VPAC2 expression 3.0 fold and PAC1 expression 10.4 fold higher compared to VPAC1 (Figure 6A). Next, we investigated if VIP or PACAP treatment could induce mRNA expression of three key markers of myelinating Schwann cells, the transcription factor Krox20, Mpz, and Mbp in primary rat Schwann cells. Schwann cells were treated with VIP or PACAP (100 nM) every 24 h for 3 days with cAMP treatment used as a positive control (Morgan et al., 1991; Monje et al., 2006). At the mRNA level, both VIP and PACAP significantly increased the expression of Krox20, Mpz, and Mbp (Figure 6F). This indicates that both VIP and PACAP may function to induce remyelination during peripheral nerve regeneration, however, PACAP appears to have a much stronger ability than VIP in driving Krox20, Mbp, and Mpz expression (Figure 6F). To investigate which receptor may be responsible for promoting Schwann cell myelination, Schwann cells were treated with VPAC1, VPAC2, and PAC1 specific agonists every 24 h for 3 days. All three receptor-specific agonists significantly increased Krox20, Mbp, and Mpz mRNA levels, indicating that this VIP and PACAP function in Schwann cells may not be receptor specific (Figure 6G). However, selective activation of PAC1 induced the biggest increase of Krox-20 and Mbp expression in Schwann cells (Figure 6G). We further used western blotting and measured Mpz and Mbp protein levels in Schwann cells after VIP, PACAP or VPAC1, VPAC2, and PAC1 specific agonist treatment. This showed that VIP, PACAP, VPAC1, VPAC2, and PAC1 specific agonists are all able to increase Mpz and Mbp protein expression (Figures 6B–E). The treatment showed that signaling through VPAC2 and PAC1 receptors has a much stronger ability to increase Mpz and Mbp protein expression in Schwann cells (Figures 6B–E). Taken together, both VIP and PACAP are able to induce Krox20, Mbp, and Mpz expression in Schwann cells, while PACAP appears to have a much stronger ability in promoting Krox20, Mbp, and Mpz expression, and VPAC2 and PAC1 receptor are the major receptors mediating their function in Schwann cells to regulate myelination.

### VIP and PACAP Inhibit the Release of Pro-inflammatory Cytokines in Schwann Cells Induced by PolyI:C and LPS

After peripheral nerve injury, Schwann cells in the distal nerve stump release pro-inflammatory cytokines such as tumor necrosis factor α (TNFα), Interleukin 6 (IL6), Interleukin 1 alpha (IL1α), Interleukin 1 beta (IL1β), and monocyte chemoattractant protein-1 (MCP-1) to recruit macrophages for clearance of both myelin and axonal debris (Martini et al., 2008; Zigmond and Echevarria, 2019). Pro-inflammatory cytokine production has to be carefully controlled in order to prevent excessive macrophage recruitment. VIP and PACAP have well characterized functions on macrophages to inhibit pro-inflammatory cytokine expression (Ganea and Delgado, 2002). To study if VIP and PACAP could inhibit pro-inflammatory cytokine expression in Schwann cells, we used both polyinosinic-polycytidylic acid (PolyI:C) and lipopolysaccharide (LPS) to induce pro-inflammatory cytokine expression in primary rat Schwann cells. Schwann cells express high levels of the toll-like receptors 3 and 4 (TLR3, TLR4), and PolyI:C activates TLR3 while LPS activates TLR4 in Schwann cells to induce pro-inflammatory cytokine expression (Goethals et al., 2010).

**FIGURE 7:**
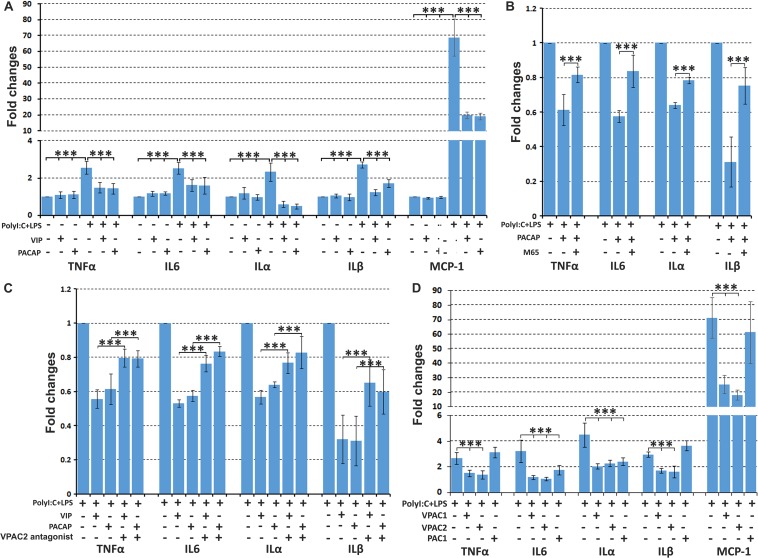
VIP and PACAP inhibit pro-inflammatory cytokine expression in cultured Schwann cells. **(A)** VIP and PACAP inhibit Poly:IC and lipopolysaccharide (LPS) induction of TNFα, IL6, ILα, ILβ, and MCP-1 expression in Schwann cells. **(B)** PAC1 receptor antagonist (M65) pre-treatment (1 h before adding PACAP) inhibits PACAP function. **(C)** VPAC2 receptor antagonist pre-treatment (1 h before adding VIP and PACAP) inhibits VIP and PACAP function. **(D)** VPAC1 and VPAC2 receptor specific agonist inhibits TNFα, IL6, ILα, ILβ, and MCP-1 expression in Schwann cells induced by PolyI:C and LPS stimulation. Note that PAC1 receptor specific agonist inhibits IL6 and ILα but not TNFα, ILβ, and MCP-1 expression. All samples were normalized to GAPDH and control samples were normalized to 1. *n* = 4. ^∗∗∗^*P* < 0.001.

As expected, PolyI:C (10 μg/ml) and LPS (100 ng/ml) treatment significantly induced TNFα, IL6, IL1α, IL1β, and MCP-1 expression in primary rat Schwann cells compared to untreated Schwann cells (Figure 7A). VIP and PACAP treatment on un-stimulated Schwann cells had no effect on TNFα, IL6, ILα, ILβ, and MCP-1 expression (Figure 7A). Adding VIP or PACAP together with PolyI:C and LPS significantly reduced the induction of TNFα, IL6, ILα, ILβ, and MCP-1 by PolyI:C, and LPS in Schwann cells (Figure 7A). In contrast, adding VPAC2 and PAC1 receptor antagonists 1 h before VIP and PACAP treatment inhibited this VIP and PACAP function (Figures 7B,C). To investigate which receptors may be responsible for VIP and PACAP inhibiting pro-inflammatory cytokine expression in Schwann cells, Schwann cells were treated with receptor specific agonists together with PolyI:C and LPS. qRT-PCR results showed that VPAC1 and VPAC2 specific agonists significantly inhibited PolyI:C and LPS-induced TNFα, IL6, ILα, ILβ, and MCP-1 expression (Figure 7D), but a PAC1 specific agonist had no effect (Figure 7D). Thus, VIP and PACAP appear to inhibit pro-inflammatory cytokine expression through VPAC1 and VPAC2 receptors on Schwann cells.

### VIP and PACAP Inhibit the Release of Pro-inflammatory Cytokines in Sciatic Nerve Explants

**FIGURE 8:**
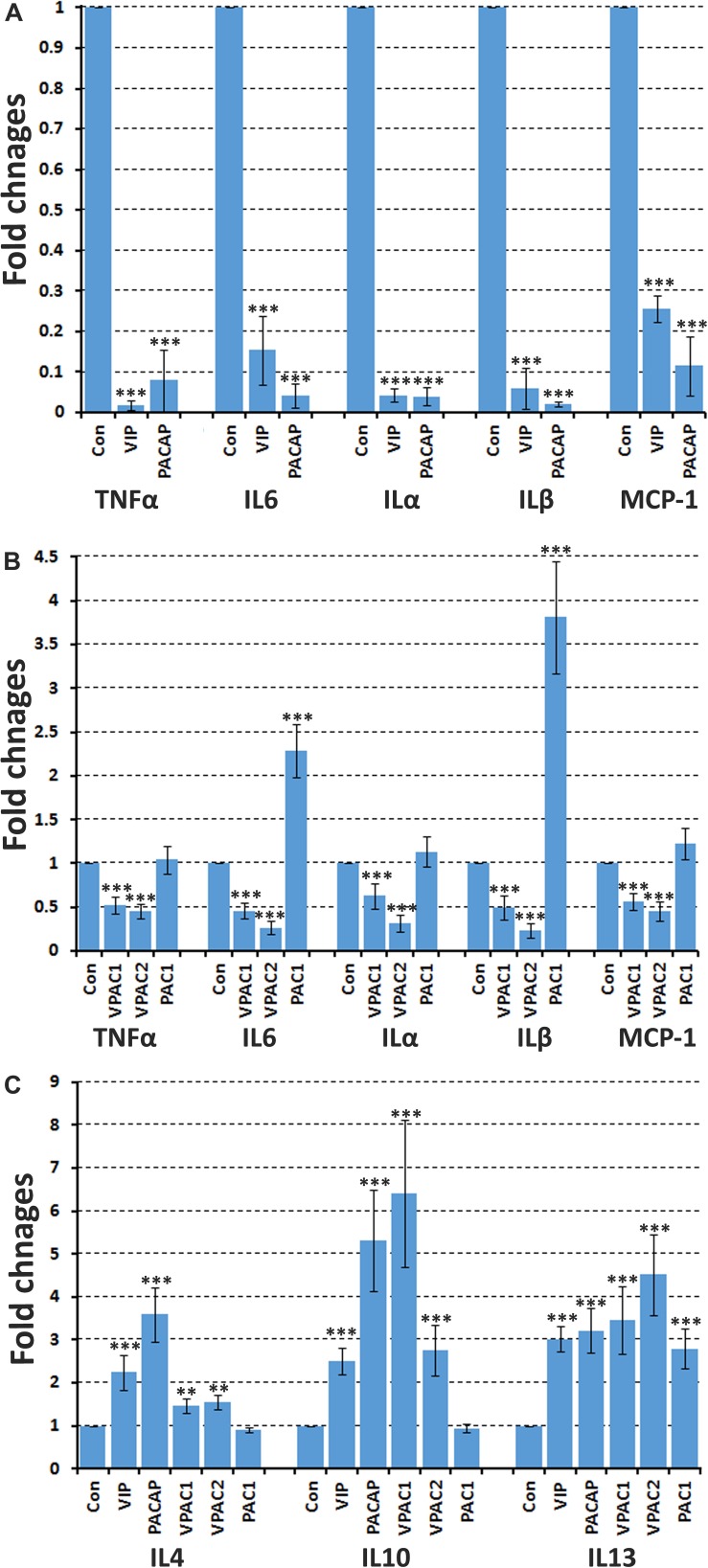
VIP and PACAP regulate pro-inflammatory and anti-inflammatory cytokine expression in nerve explants. **(A)** 24 h treatment with VIP or PACAP inhibit TNFα, IL6, ILα, ILβ, and MCP-1 expression in cultured sciatic nerve explants. Sciatic nerve explants were cultured from uninjured mouse sciatic nerve. **(B)** 24 h treatment with VPAC1 or VPAC2 receptor-specific agonist treatment inhibits TNFα, IL6, ILα, ILβ, and MCP-1 expression in cultured sciatic nerve explants. In contrast, PAC1 receptor specific agonist treatment induces IL6 and ILβ expression. Sciatic nerve explants were cultured from uninjured mouse sciatic nerve. **(C)** 24 h treatment with VIP, PACAP or receptor specific agonists induce anti-inflammatory cytokines IL4, IL10, and IL13 expression in nerve explants. Nerve explants were cultured from the distal nerve stump at 10 days post-transection injury. All samples were normalized to GAPDH and control samples were made relative to 1. *n* = 3. ^∗∗^*P* < 0.01, ^∗∗∗^*P* < 0.001. 27 mice were used in **(A,B)** for nerve explant culture, and 54 mice were used in **(C)** for nerve explant culture.

Peripheral nerve injury rapidly triggers secretion of pro-inflammatory cytokines such as TNFα, IL6, ILα, ILβ, and MCP-1 by Schwann cells in the distal nerve stump to recruit macrophages for myelin and axonal debris clearance (Martini et al., 2008; Zigmond and Echevarria, 2019). The pro-inflammatory cytokine expression in the distal nerve peaks at 24 h following peripheral nerve injury (Rotshenker, 2011). To test whether VIP and PACAP could inhibit pro-inflammatory cytokine secretion in the injured peripheral nerve, we used mouse adult sciatic nerve explants and incubated with VIP and PACAP peptides. The nerve dissection and explant culture triggers the injury response and induces TNFα, IL6, ILα, ILβ, and MCP-1 secretion from Schwann cells of the nerve explants (Figure 8A). After 24 h of VIP and PACAP incubation, mRNA was extracted for subsequent qRT-PCR analysis to measure TNFα, IL6, ILα, ILβ, and MCP-1 expression. qRT-PCR results showed that both VIP and PACAP significant inhibited TNFα, IL6, ILα, ILβ, and MCP-1 expression in sciatic nerve explants (Figure 8A). To determine which receptor VIP and PACAP may be acting through to decrease pro-inflammatory cytokine expression in the nerve explants, the same experiments were performed but nerve explants were incubated with receptor-specific agonists. Treatment with each receptor-specific agonist showed that stimulation with VPAC1 and VPAC2 specific agonists significantly down-regulated the expression of pro-inflammatory cytokines investigated (Figure 8B). Treatment with a PAC1-specific agonist had no effect upon TNFα and ILα expression (Figure 8B), but, to our surprise, the PAC1-specific agonist significantly up-regulated IL6, ILβ, and MCP-1 expression in sciatic nerve explants (Figure 8B). As PACAP up-regulation reaches its peak on day 2 following injury, which is much earlier than the peak of VIP expression on day 7, the regulation of IL6, ILβ, and MCP-1 by PACAP in nerve explants indicates that PACAP may promote early pro-inflammatory cytokine production in the sciatic nerve following injury. In line with above results for IL6, ILβ, and MCP-1 production in cultured Schwann cells, VIP and PACAP appear to act through VPAC1 and VPAC2 receptors in Schwann cells in the nerve explants to inhibit pro-inflammatory cytokine expression.

### VIP and PACAP Enhance Anti-inflammatory Cytokine Production in Mouse Nerve Explants

Macrophages in the distal nerve stump secrete anti-inflammatory cytokines such as IL4, IL10, and IL13 to promote peripheral nerve regeneration (Martini et al., 2008; Ydens et al., 2012; Zigmond and Echevarria, 2019). Macrophages infiltrate the injured nerves from day 3 and the total macrophage number within the injured nerve peaks between day 10 and day 14 post-injury and decreases thereafter (Martini et al., 2008; Zigmond and Echevarria, 2019). A previous study showed that 20.3% cells are macrophages in the mouse distal sciatic nerve at 10 days following nerve transection (Stierli et al., 2018). To investigate if VIP and PACAP could act on mouse distal nerve and promote anti-inflammatory cytokine expression, we treated nerve explants taken from the distal nerve stump 10 days post-injury with VIP and PACAP for 24 h and then investigated the expression of anti-inflammatory cytokines IL4, IL10, and IL13. qRT-PCR results showed that both VIP and PACAP are able to significantly increase the expression of anti-inflammatory cytokines IL4, IL10, and IL13 in distal nerve explants (Figure 8C). To determine which receptor VIP and PACAP may be acting through to increase IL4, IL10, and IL13 expression in the nerve explants, we incubated the nerve explants with receptor specific agonists and then measured the levels of IL4, IL10, and IL13 expression. We found that VPAC1 and VPAC2 activation significantly up-regulated the expression of IL4, IL10, and IL13. However, PAC1 stimulation only up-regulated IL13, it had no effect on IL10 and IL4 expression (Figure 8C), indicating that VPAC1 and VPAC2 are the major receptors increasing anti-inflammatory expression in the distal nerve explants.

## Discussion

Vasoactive Intestinal Peptide and PACAP are regeneration-associated neuropeptides that are up-regulated in motor, sensory, and sympathetic neurons following peripheral nerve injury (Xiao et al., 2002; Armstrong et al., 2003; Navarro et al., 2007). Their up-regulation in motor neurons is detectable 6 h after peripheral nerve injury and both reach more than a 20-fold peak increase (Zhou et al., 1999; Xiao et al., 2002; Armstrong et al., 2003). Although previous studies have been focused upon studying the autocrine effect of VIP and PACAP on neuronal survival and axon outgrowth during peripheral nerve regeneration, these studies have also revealed that the increase of VIP and PACAP expression in neurons was accompanied by a decrease in expression of their receptors (Zhou et al., 1999). For instance, PAC1 mRNA expression was decreased to 50% in motor neurons by 6 h after facial nerve axotomy comparing to uninjured site, and by 1 week it had decreased to about 25%. Thereafter, the level of PAC1 mRNA remained low at about 20–25% for up to 30 days to the levels of control animal motor neurons (Zhou et al., 1999; Waschek et al., 2000). The down-regulation of PAC1 has been explained as G protein-coupled receptor desensitization in response to ligand binding (Zhou et al., 1999; Waschek et al., 2000). Our new data reveals a plausible reason for the receptor down-regulation in neurons, namely to allow VIP and PACAP to execute their important function on Schwann cells and macrophages in the distal nerve stump during regeneration. Using qRT-PCR and western blot methods, we confirmed that VPAC1, VAPC2, and PAC1 are all up-regulated in the distal nerve stump after peripheral nerve injury. By using cell-specific markers, we further showed that the receptor proteins are highly expressed in Schwann cells and infiltrating macrophages of the distal nerve stump.

Previous reports have shown that VPAC1, VPAC2, and PAC1 are expressed in cultured Schwann cells and schwannoma cells (Zhang et al., 1996; Castorina et al., 2008, 2014; Lee et al., 2009). Lee et al. (2009) compared VPAC1 and VPAC2 expression levels in cultured Schwann cells and showed that Schwann cells expressed higher levels of VPAC2 mRNA than VPAC1 mRNA. In line with these findings, our results also showed that the VPAC2 mRNA level is 3 fold higher than VPAC1 mRNA expression in Schwann cells. We also showed that PAC1 has the highest level of expression in Schwann cells (Figure 8A). Zhang et al. (1996) were the first to report a VIP function on Schwann cells. They showed that VIP treatment on cultured primary Schwann cells induced laminin synthesis (Zhang et al., 1996). More than 10 years later, Lee et al. (2009) published the second report showing that VIP has a direct effect upon Schwann cells. Lee et al. (2009) reported that VIP pre-treatment inhibited LPS-induced nitric oxide (NO) synthase gene expression and NO production in Schwann cells. In addition to these two papers studying the direct effect of VIP on primary Schwann cells, two other reports have shown that the schwannoma cell lines CRL-2768 and RT4-P6D2T both express VPAC2 and PAC1 receptors (Castorina et al., 2008, 2014). VIP and PACAP treatment on rat RT4 schwannoma cells not only prevented cell apoptosis but also induced myelin protein expression (Castorina et al., 2008, 2014). *In vivo* studies also showed that injection of VIP into the mouse sciatic nerve gap following nerve transection and delivery of VIP-expressing mesenchymal stem cells into a nerve guidance conduit for rat sciatic nerve gap repair accelerated Schwann cell re-myelination (Zhang et al., 2002; Hernandez-Cortes et al., 2014). Binding of VIP and PACAP to their receptors increases intracellular cAMP (Harmar et al., 1998; Castorina et al., 2014), and cAMP is a key signaling molecule to induce Schwann cell myelination during peripheral nerve development (Jessen and Mirsky, 2005; Monk et al., 2009). Thus, VIP and PACAP could be important signals to promote peripheral nerve re-myelination during regeneration. Their effects on Schwann cell re-myelination could be mediated by increase intracellular cAMP levels. Previous studies also showed that binding of PACAP to PAC1 had a much stronger ability to induce cAMP production than binding to VPAC1 and VPAC2 receptors (Vaudry et al., 2009). In light of this, then it is perhaps not surprising that we observed PAC1 as the most effective at inducing Krox20, Mbp, and Mpz expression in Schwann cells. Thus, it appears that PACAP has more important role in Schwann cell re-myelination although VIP does still apparently have the ability to promote re-myelination during peripheral nerve regeneration.

After peripheral nerve injury, large numbers of macrophages infiltrate into the distal nerve stump to clear axonal and myelin debris; infiltrated macrophages also release pro-inflammatory cytokines to recruit more macrophages to the distal nerve (Ip et al., 2006; Zigmond, 2012; Zigmond and Echevarria, 2019). The rapid inflammatory response after peripheral nerve injury must be carefully controlled in order to prevent excessive macrophage recruitment and unnecessary inflammation and tissue damage. Currently, signals that are required to balance the pro-inflammatory cytokines and anti-inflammatory cytokines production in the distal nerve stump have not been well studied (Martini et al., 2008). In the later stages of regeneration, macrophages in the distal nerve stump undergo a stage transition to completely down-regulate pro-inflammatory cytokines production and further up-regulate anti-inflammatory cytokines expression (Fry et al., 2007). Again, signals that regulate macrophage stage transition in the peripheral nerves during regeneration have not been characterized. Staining on mouse distal nerve, at 7 days post-injury, showed that macrophages within the distal nerve stump express VPAC1, VPAC2, and PAC1. Incubation of distal nerve explants, taken at 10 days post-injury, with VIP or PACAP for 24 h up-regulated anti-inflammatory cytokine IL4, IL10, and IL13 expression. Thus, our studies have identified VIP and PACAP as key signaling molecules to balance pro-inflammatory and anti-inflammatory cytokine production and potentially macrophage stage transition in the distal nerve stump. These are key steps for the resolution of the inflammatory response of the injured peripheral nerve and re-establishment of tissue homeostasis. Indeed, a study in the PACAP knockout animals not only showed that axon regeneration following injury is reduced, but also found that pro-inflammatory cytokine down-regulation is delayed and anti-inflammatory cytokine production is impaired in the distal nerve stump following injury (Armstrong et al., 2008).

Interestingly, the up-regulation of VIP and PACAP in neurons was found to be regulated by cytokines present in the distal nerve stump (Habecker et al., 2009; Zigmond, 2012). Given the well characterized role of VIP and PACAP in macrophage stage transition in other tissue and organs together with a large amount of VIP and PACAP secretion by the regenerating axons to the distal nerve stump, this implies a possible feedback mechanism that, upon the up-regulation of pro-inflammatory cytokines in the distal nerve stump, they not only recruit macrophages to the distal nerve stump but also stimulate VIP and PACAP secretion from neurons. Subsequently, VIP and PACAP act as immunomodulators on infiltrating macrophages to balance pro-inflammatory and anti-inflammatory cytokines production in the distal nerve stump. In support of this idea, the time and the peak of VIP expression not only coincides with macrophage accumulation in the distal nerve stump during regeneration, but also matches with the kinetics of IL-10 production (a classic anti-inflammatory factor) in the distal nerve stump. IL-10 expression starts to increase considerably in the distal nerve stump 4 days post-injury, peaks at day 7 and remains elevated during the course of regeneration (Be’eri et al., 1998).

Peripheral nerve injury triggers TNFα, MCP-1, ILα, ILβ, and IL6 expression in Schwann cells and they peak at 24 h following injury (Rotshenker, 2011). Increasing evidence shows that there are remarkable similarities between inherited peripheral neuropathies and peripheral nerve trauma in term of inflammatory response although the former is chronic and the latter is acute (Martini et al., 2013; Klein and Martini, 2016). In pathological situations such as Charcot-Marie-Tooth (CMT) disease, acute inflammatory demyelinating polyneuropathy (AIDP), and chronic inflammatory demyelinating polyneuropathy (CIDP), Schwann cells also release pro-inflammatory cytokines and recruit macrophages to the peripheral nerves. Macrophages are the major immune cells to be found in the peripheral nerves of animal models for CMT diseases (Fledrich et al., 2012; Martini et al., 2013). In response to chemokines released by Schwann cells in CMT animal models, macrophages enter into the peripheral nerves and phagocytose myelin to leave the demyelinated axons intact and, thus, they strongly contribute to the pathogenesis of CMT disorders (Ip et al., 2006).

Investigations in human nerve biopsies have revealed that abnormal macrophage recruitment plays a key role in the demyelination process in neuropathies such as CMT, AIDP, and CIDP diseases (Martini et al., 2013; Klein and Martini, 2016). Inherited peripheral neuropathies in humans are still incurable and lead to muscle wasting, sensory dysfunction and progressive disability (Fledrich et al., 2012). As such, the development of novel therapeutic approaches becomes important. In this study, we show that VIP and PACAP treatment not only inhibits TNFα, MCP-1, ILα, ILβ, and IL6 expression in Schwann cells induced by Poly:IC and LPS stimulation but also significantly reduced TNFα, MCP-1, ILα, ILβ, and IL6 expression in nerve explants. Our findings from this study have indicated the potential for using exogenous VIP and PACAP through VPAC1 and VPAC2 receptors to inhibit the release of pro-inflammatory cytokines by Schwann cells in such pathological situations. Thus, further investigation of VIP and PACAP immunomodulatory function on CMT mouse models could potentially develop VIP and PACAP as novel molecules for the treatment of peripheral neuropathies.

We show here that up-regulated VIP and PACAP could act on both Schwann cells and macrophages in the distal nerve stump and potentially regulate peripheral nerve regeneration. Although both VIP and PACAP were up-regulated in neurons following peripheral nerve injury, PACAP reaches its peak expression at day 2 while VIP reaches its peak expression at day 7 (Armstrong et al., 2003; Navarro et al., 2007). Their peak difference indicates that VIP and PACAP may execute distinct function in the distal nerve stump at different time points during regeneration. Our treatment with PAC1 specific agonist significantly up-regulated IL6, ILβ and MCP-1 expression in sciatic nerve explants (Figure 8B). This result indicated that PACAP may have the ability to promote early pro-inflammatory cytokine production in the sciatic nerve following injury. However, activation of PAC1 has no effect on the release of pro-inflammatory cytokine in cultured Schwann cells (Figure 7B), indicating that PACAP may act on a different cell type in the sciatic nerve explants and promote pro-inflammatory cytokine release. About 8% cells in the peripheral nerves are known to be resident macrophages and their activation contributes significantly to the early release of pro-inflammatory cytokine following peripheral nerve injury (Stierli et al., 2018; Zigmond and Echevarria, 2019). We showed that macrophages in the mouse sciatic nerve express PAC1 the receptor (Figure 5), thus, PACAP peaking at day 2 following injury may have an important function by acting on resident macrophages to promote early pro-inflammatory cytokine production. In contrast, the peak expression of VIP on day 7 following peripheral nerve injury well matches with the time point of pro-inflammatory cytokine down-regulation. Therefore, VIP could have more a important function than PACAP in terms of the resolution of the distal nerve inflammatory response.

Taken together, in this study, we showed that VPAC1, VPAC2, and PAC1 are up-regulated in Schwann cells and macrophages of the distal nerve stump after peripheral nerve injury. Neuron secreted VIP and PACAP could bind to these receptors on Schwann cells and macrophages in the distal stump to execute distinct functions at different stages of regeneration. In the early stage of peripheral nerve injury, VIP and PACAP could balance pro-inflammatory cytokine production and prevent unnecessary macrophage recruitment. In the later stage of regeneration, VIP and PACAP may down-regulate the expression of pro-inflammatory cytokines and up-regulate the production of anti-inflammatory cytokines in macrophages to trigger the macrophage stage transition and eventually terminate the inflammatory response in the distal nerve stump. In the later stages of peripheral nerve regeneration, VIP and PACAP may also have important function in promoting Schwann cell re-myelination.

## Data Availability Statement

The datasets generated for this study are available on request to the corresponding author.

## Ethics Statement

The animal study was reviewed and approved by the University of Plymouth Animal Welfare and Ethical Review Board.

## Author Contributions

XD designed the research. PW, QM, YL, and NM performed the experiments and analyzed the data. XD and DP wrote the manuscript.

## Conflict of Interest

The authors declare that the research was conducted in the absence of any commercial or financial relationships that could be construed as a potential conflict of interest.

## References

[B1] Armstrong B. D. Abad C. Chhith S. Cheling-Lau G. Hajji O. E. Noblita H. (2008). Impaired nerve regeneration and enhanced neuroinflarnmatory response in mice lacking pituitary adenylyl cyclase activating peptide. *Neuroscience* 151 63–73. 10.1016/j.neuroscience.200.09.084 18055122 PMC2245872

[B2] Armstrong B. D. Hu Z. Abad C. Yamamoto M. Rodriguez W. I. Cheng J. (2003). Lymphocyte regulation of neuropeptide gene expression after neuronal injury. *J. Neurosci. Res.* 74 240–247. 10.1002/jnr.10750 14515353

[B3] Be’eri H. Reichert F. Saada A. Rotshenker S. (1998). The cytokine network of wallerian degeneration: IL-10 and GM-CSF. *Eur. J. Neurosci.* 10 2707–2713. 10.1046/j.1460-9568.1998.00277.x 9767400

[B4] Brockes J. P. Fields K. L. Raff M. C. (1979). Studies on cultured rat Schwann cells. I. Establishment of purified populations from cultures of peripheral nerve. *Brain Res.* 165 105–118. 10.1016/0006-8993(79)90048-9 371755

[B5] Carr L. Parkinson D. B. Dun X. P. (2017). Expression patterns of Slit and Robo family members in adult mouse spinal cord and peripheral nervous system. *PLoS One* 12:e0172736. 10.1371/journal.pone.0172736 28234971 PMC5325304

[B6] Castorina A. Scuderi S. D’Amico A. G. Drago F. D’Agata V. (2014). PACAP and VIP increase the expression of myelin-related proteins in rat schwannoma cells: involvement of PAC1/VPAC2 receptor-mediated activation of PI3K/Akt signaling pathways. *Exp. Cell. Res.* 322 108–121. 10.1016/j.yexcr.2013.11.003 24246222

[B7] Castorina A. Tiralongo A. Giunta S. Carnazza M. L. Rasi G. D’Agata V. (2008). PACAP and VIP prevent apoptosis in schwannoma cells. *Brain Res.* 1241 29–35. 10.1016/j.brainres.2008.09.035 18835258

[B8] Delgado M. Munoz-Elias E. J. Martinez C. Gomariz R. P. Ganea D. (1999). VIP and PACAP38 modulate cytokine and nitric oxide production in peritoneal macrophages and macrophage cell lines. *Ann. N. Y. Acad. Sci.* 897 401–414. 10.1111/j.1749-6632.1999.tb07909.x 10676466

[B9] Delgado M. Pozo D. Ganea D. (2004). The significance of vasoactive intestinal peptide in immunomodulation. *Pharmacol. Rev.* 56 249–290. 10.1124/pr.56.2.7 15169929

[B10] Dun X. P. Carr L. Woodley P. K. Barry R. W. Drake L. K. Mindos T. (2019). Macrophage-derived Slit3 controls cell migration and axon pathfinding in the peripheral nerve bridge. *Cell Rep.* 26:1458-1472.e4c. 10.1016/j.celrep.2018.12.081 30726731 PMC6367597

[B11] Fledrich R. Stassart R. M. Sereda M. W. (2012). Murine therapeutic models for Charcot-Marie-Tooth (CMT) disease. *Br. Med. Bull.* 102 89–113. 10.1093/bmb/lds010 22551516

[B12] Fry E. J. Ho C. David S. (2007). A role for nogo receptor in macrophage clearance from injured peripheral nerve. *Neuron* 53 649–662. 10.1016/j.neuron.2007.02.009 17329206

[B13] Ganea D. Delgado M. (2002). Vasoactive intestinal peptide (VIP) and pituitary adenylate cyclase-activating polypeptide (PACAP) as modulators of both innate and adaptive immunity. *Crit. Rev. Oral. Biol. Med.* 13 229–237. 12090463 10.1177/154411130201300303

[B14] Goethals S. Ydens E. Timmerman V. Janssens S. (2010). Toll-Like receptor expression in the peripheral nerve. *Glia* 58 1701–1709. 10.1002/glia.21041 20578041

[B15] Habecker B. A. Sachs H. H. Rohrer H. Zigmond R. E. (2009). The dependence on gp130 cytokines of axotomy induced neuropeptide expression in adult sympathetic neurons. *Dev. Neurobiol.* 69 392–400. 10.1002/dneu.20706 19280647 PMC2721278

[B16] Harmar A. J. Arimura A. Gozes I. Journot L. Laburthe M. Pisegna J. R. (1998). International union of pharmacology. XVIII. nomenclature of receptors for vasoactive intestinal peptide and pituitary adenylate cyclase-activating polypeptide. *Pharmacol. Rev.* 50 265–270. 9647867 PMC6721840

[B17] Hernandez-Cortes P. Toledo-Romero M. A. Delgado M. Sanchez-Gonzalez C. E. Martin F. Galindo-Moreno P. (2014). Peripheral nerve reconstruction with epsilon-caprolactone conduits seeded with vasoactive intestinal peptide gene-transfected mesenchymal stem cells in a rat model. *J. Neural. Eng.* 11:046024. 10.1088/1741-2560/11/4/046024 25024301

[B18] Ip C. W. Kroner A. Bendszus M. Leder C. Kobsar I. Fischer S. (2006). Immune cells contribute to myelin degeneration and axonopathic changes in mice overexpressing proteolipid protein in oligodendrocytes. *J. Neurosci.* 26 8206–8216. 10.1523/JNEUROSCI.1921-06.2006 16885234 PMC6673777

[B19] Jessen K. R. Mirsky R. (2005). The origin and development of glial cells in peripheral nerves. *Nat. Rev. Neurosci.* 6 671–682. 10.1038/nrn1746 16136171

[B20] Klein D. Martini R. (2016). Myelin and macrophages in the PNS: an intimate relationship in trauma and disease. *Brain Res.* 1641(Pt A), 130–138. 10.1016/j.brainres.2015.11.033 26631844

[B21] Lee H. Park K. Kim J. S. Lee S. J. (2009). Vasoactive intestinal peptide inhibits toll-like receptor 3-induced nitric oxide production in schwann cells and subsequent sensory neuronal cell death in vitro. *J. Neurosci. Res.* 87 171–178. 10.1002/jnr.21820 18683246

[B22] Lioudyno M. Skoglosa Y. Takei N. Lindholm D. (1998). Pituitary adenylate cyclase-activating polypeptide (PACAP) protects dorsal root ganglion neurons from death and induces calcitonin gene-related peptide (CGRP) immunoreactivity in vitro. *J. Neurosci. Res.* 51 243–256. 10.1002/(sici)1097-4547(19980115)51:2<243::aid-jnr13>3.3.co;2-n 9469578

[B23] Livak K. J. Schmittgen T. D. (2001). Analysis of relative gene expression data using real-time quantitative PCR and the 2(-Delta Delta C(T)) Method. *Methods* 25 402–408. 10.1006/meth.2001.1262 11846609

[B24] Ma T. C. Willis D. E. (2015). What makes a RAG regeneration associated? *Front. Mol. Neurosci.* 8:43. 10.3389/fnmol.2015.00043 26300725 PMC4528284

[B25] Mallon B. S. Shick H. E. Kidd G. J. Macklin W. B. (2002). Proteolipid promoter activity distinguishes two populations of NG2-positive cells throughout neonatal cortical development. *J. Neurosci.* 22 876–885. 10.1523/jneurosci.22-03-00876.2002 11826117 PMC6758537

[B26] Martini R. Fischer S. Lopez-Vales R. David S. (2008). Interactions between Schwann cells and macrophages in injury and inherited demyelinating disease. *Glia* 56 1566–1577. 10.1002/glia.20766 18803324

[B27] Martini R. Klein D. Groh J. (2013). Similarities between inherited demyelinating neuropathies and wallerian degeneration: an old repair program may cause myelin and axon perturbation under nonlesion conditions. *Am. J. Pathol.* 183 655–660. 10.1016/j.ajpath.2013.06.002 23831295

[B28] Miyata A. Arimura A. Dahl R. R. Minamino N. Uehara A. Jiang L. (1989). Isolation of a novel-38 residue-hypothalamic polypeptide which stimulates adenylate-cyclase in pituitary-cells. *Biochem. Biophys. Res. Commun.* 164 567–574. 10.1016/0006-291x(89)91757-9 2803320

[B29] Monje P. V. Bartlett Bunge M. Wood P. M. (2006). Cyclic AMP synergistically enhances neuregulin-dependent ERK and Akt activation and cell cycle progression in Schwann cells. *Glia* 53 649–659. 10.1002/glia.20330 16470843

[B30] Monk K. R. Naylor S. G. Glenn T. D. Mercurio S. Perlin J. R. Dominguez C. (2009). A G protein-coupled receptor is essential for schwann cells to initiate myelination. *Science* 325 1402–1405. 10.1126/science.1173474 19745155 PMC2856697

[B31] Morgan L. Jessen K. R. Mirsky R. (1991). The effects of cAMP on differentiation of cultured schwann cells: progression from an early phenotype (04+) to a myelin phenotype (P0+, GFAP-, N- CAM-, NGF-receptor-) depends on growth inhibition. *J. Cell Biol.* 112 457–467. 10.1083/jcb.112.3.457 1704008 PMC2288828

[B32] Navarro X. Vivo M. Valero-Cabre A. (2007). Neural plasticity after peripheral nerve injury and regeneration. *Prog. Neurobiol.* 82 163–201. 10.1016/j.pneurobio.2007.06.005 17643733

[B33] Reimer M. Moller K. Sundler F. Hannibal J. Fahrenkrug J. Kanje M. (1999). Increased expression, axonal transport and release of pituitary adenylate cyclase-activating polypeptide in the cultured rat vagus nerve. *Neuroscience* 88 213–222. 10.1016/s0306-4522(98)00240-1 10051202

[B34] Rotshenker S. (2011). Wallerian degeneration: the innate-immune response to traumatic nerve injury. *J. Neuroinflamm.* 8:109. 10.1186/1742-2094-8-109 21878125 PMC3179447

[B35] Said S. I. Mutt V. (1970). Potent peripheral and splanchnic vasodilator peptide from normal gut. *Nature* 225 863–864. 10.1038/225863a0 5415118

[B36] Said S. I. Mutt V. (1972). Isolation from porcine-intestinal wall of a vasoactive octacosapeptide related to secretin and to glucagon. *Eur. J. Biochem.* 28 199–204. 10.1111/j.1432-1033.1972.tb01903.x 5069712

[B37] Stierli S. Napoli I. White I. J. Cattin A. L. Cabrejos A. M. Calavia N. G. (2018). The regulation of the homeostasis and regeneration of peripheral nerve is distinct from the CNS and independent of a stem cell population. *Development* 145:dev170316. 10.1242/dev.170316 30413560 PMC6307893

[B38] Vaudry D. Falluel-Morel A. Bourgault S. Basille M. Burel D. Wurtz O. (2009). Pituitary adenylate cyclase-activating polypeptide and its receptors: 20 years after the discovery. *Pharmacol. Rev.* 61 283–357. 10.1124/pr.109.001370 19805477

[B39] Waschek J. A. (2013). VIP and PACAP: neuropeptide modulators of CNS inflammation, injury, and repair. *Br. J. Pharmacol.* 169 512–523. 10.1111/bph.12181 23517078 PMC3682700

[B40] Waschek J. A. Dicicco-Bloom E. M. Lelievre V. Zhou X. Hu Z. (2000). PACAP action in nervous system development, regeneration, and neuroblastoma cell proliferation. *Ann. N. Y. Acad. Sci.* 921 129–136. 10.1111/j.1749-6632.2000.tb06959.x 11193816

[B41] Xiao H. S. Huang Q. H. Zhang F. X. Bao L. Lu Y. J. Guo C. (2002). Identification of gene expression profile of dorsal root ganglion in the rat peripheral axotomy model of neuropathic pain. *Proc. Natl. Acad. Sci. U.S.A.* 99 8360–8365. 10.1073/pnas.122231899 12060780 PMC123072

[B42] Ydens E. Cauwels A. Asselbergh B. Goethals S. Peeraer L. Lornet G. (2012). Acute injury in the peripheral nervous system triggers an alternative macrophage response. *J. Neuroinflamm.* 9:176. 10.1186/1742-2094-9-176 22818207 PMC3419084

[B43] Zhang Q. L. Lin P. X. Shi D. Xian H. Webster H. D. (1996). Vasoactive intestinal peptide: mediator of laminin synthesis in cultured Schwann cells. *J. Neurosci. Res.* 43 496–502. 10.1002/(sici)1097-4547(19960215)43:4<496::aid-jnr11>3.0.co;2-0 8699536

[B44] Zhang Q. L. Liu J. Lin P. X. Webster H. (2002). Local administration of vasoactive intestinal peptide after nerve transection accelerates early myelination and growth of regenerating axons. *J. Peripher. Nerv. Syst.* 7 118–127. 10.1046/j.1529-8027.2002.02018.x 12090298

[B45] Zhou X. Rodriguez W. I. Casillas R. A. Ma V. Tam J. Hu Z. (1999). Axotomy-induced changes in pituitary adenylate cyclase activating polypeptide (PACAP) and PACAP receptor gene expression in the adult rat facial motor nucleus. *J. Neurosci. Res.* 57 953–961. 10.1002/(sici)1097-4547(19990915)57:6<953::aid-jnr21>3.0.co;2-r 10467267

[B46] Zigmond R. E. (2012). Cytokines that promote nerve regeneration. *Exp. Neurol.* 238 101–106. 10.1016/j.expneurol.2012.08.017 22981450 PMC4085665

[B47] Zigmond R. E. Echevarria F. D. (2019). Macrophage biology in the peripheral nervous system after injury. *Prog. Neurobiol.* 173 102–121. 10.1016/j.pneurobio.2018.12.001 30579784 PMC6340791

